# Effects of Growth Hormone-Releasing Hormone (GHRH) Plasmid Treatment on the Reproductive Productivity of Sows in Primiparous and Multiparous Sow Breeds

**DOI:** 10.3390/ani14081148

**Published:** 2024-04-10

**Authors:** Min Jung Kim, Ji-Yong Park, Chang-Soo Cho, Ye Jin Yang, Ji Woong Heo, Woo H. Kim, Hu-Jang Lee, Kwang Il Park

**Affiliations:** College of Veterinary, Gyeongsang National University, Jinju 52828, Republic of Korea; min-jung95@naver.com (M.J.K.); genius9696@naver.com (J.-Y.P.); feeljcs@naver.com (C.-S.C.); yang93810@gnu.ac.kr (Y.J.Y.); hujiw7806@naver.com (J.W.H.); woohyun.kim@gnu.ac.kr (W.H.K.)

**Keywords:** growth hormone-releasing hormone, insulin-like growth factor-1, multiparous sow, primiparous sow

## Abstract

**Simple Summary:**

In the field of veterinary science and animal husbandry, a sow that has given birth more than once is considered “multiparous”. Sows are classified as nulliparous from the moment they arrive at the farm until their first farrowing. During this time, they are frequently referred to as “primiparous”. Growth hormone-releasing hormone (GHRH) plasmid administration had significant effects on serum insulin-like growth factor-1 (IGF-1) levels, birth outcomes, and weaning parameters across different sow categories, indicating its potential for enhancing reproductive performance and postnatal outcomes in sows. Specifically, it affected live births, survival rates, and weaning success without notable effects on birth weight.

**Abstract:**

The effect of growth hormone-releasing hormone (GHRH) plasmid treatment on sow reproductive performance was examined. Forty pregnant sows (three-way crossbreed: Landrace × Yorkshire × Duroc) at 85 days of gestation were included in the study and consisted of twenty primiparous and twenty multiparous sows (third parity). Sows were randomly assigned to the control and treatment groups. The treatment group received 5 mg dose of GHRH plasmid injection via electroporation, whereas the control group received a phosphate buffer solution. Reproductive indicators, including serum insulin-like growth factor-1 (IGF-1) concentration and weaned piglet data, were assessed. In the GHRH plasmid-treated group, serum IGF-1 concentration significantly increased compared with that in the control group, a trend observed in primiparous and multiparous sows. The key indicator of reproductive performance, litter size, showed that for control primiparous sows (C-PS), it was 10.90 ± 0.99 kg, while for control multiparous sows (C-MS), it was 14.00 ± 0.67 kg. Furthermore, for primiparous sows treated with GHRH plasmid (G-PS), the litter size was 11.60 ± 0.97 kg, and for multiparous sows treated with GHRH plasmid (G-MS), it was 14.00 ± 0.82 kg. The GHRH plasmid-treated group also exhibited a higher number of total births and surviving piglet numbers, along with a decrease in stillborn piglets; however, there was no significant difference in birth weight. The results suggest that GHRH plasmid treatment can enhance the reproductive performance of sows.

## 1. Introduction

The primary controllers of growth hormone production and release are the two hypothalamic hormones, growth hormone-releasing hormone (GHRH) and somatostatin [1]. They act on virtually every tissue of the body to control metabolism and growth. GHRH and somatostatin regulate growth hormone secretion in a highly precise manner by exerting opposing actions, which differ based on age, gender, and metabolic conditions. The effect of GHRH on the pituitary gland is counteracted by somatostatin, a hormone also produced by the hypothalamus, which prevents growth hormone release. GHRH is produced in the hypothalamus and stimulates the synthesis and secretion of growth hormone (GH) from the pituitary gland [2]. The pulsatile release of GHRH triggers the corresponding pulses of GH to influence various physiological processes, such as growth, cellular reproduction, and metabolism. To maintain balanced hormone production, GHRH, somatostatin, GH, and insulin-like growth factor 1 IGF-1 levels are regulated by one another.

GH stimulates the production of insulin-like growth factor 1 in the liver and other organs. Growth factor 1, also known as IGF-1, is an important peptide-like hormone that affects physiological activities [3]. It is significantly associated with growth and development and plays an important role in porcine reproduction [4]. In addition, GHRH/GH/IGF-I significantly regulates growth, development [5], pregnancy, and breastfeeding [6]. GH synthesis in the liver is triggered by the secretion of GH from the pituitary gland [7]. This dynamic interplay between IGF-1 and GH is instrumental in the regulation of various physiological functions associated with growth and development. Studies have demonstrated the occurrence of transplacental transmission of GHRH from the mother to the fetus, which can directly influence fetal development [8]. In particular, this effect extends to ovarian function and follicle development, which are important aspects of reproductive performance in female pigs [9].

In the swine production industry, sow prolificacy is an important factor. It encompasses the reproductive efficiency and prolific breeding performance of female pigs. Litter size is an important metric, which denotes the number of piglets born per sow [10], and is a direct determinant of the economic viability and sustainability of pig farming operations.

Various factors influence sow prolificacy, including genetics, nutrition, management practices, and hormonal regulation. Achieving and maintaining optimal sow prolificacy is essential for maximizing the productivity of a swine herd.

It is unclear to what degree the enhanced growth and survival of the offspring may be attributed to advancements in maternal physiology during pregnancy and nursing, as opposed to changes in the offspring [11,12]. In this study, we focused on primiparous and multiparous sow breeds to determine the role of the GHRH plasmid treatment (GHRH-T) group on sow prolificacy.

## 2. Materials and Methods

### 2.1. Experimental Animals

**Figure 1:**
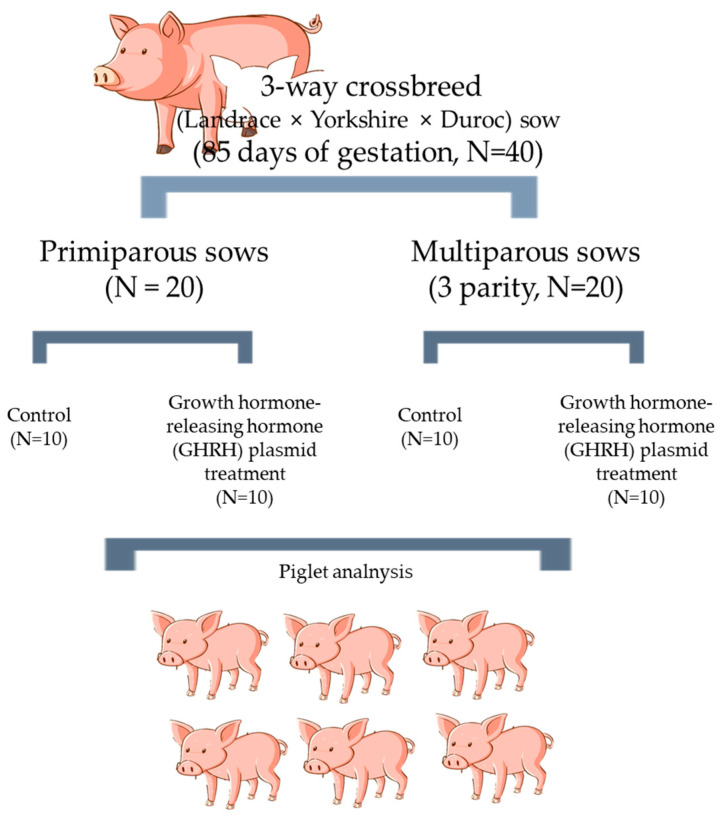
Illustration depicting the systematic process used to administer GHRH to sows.

This study was conducted at a farm located in Gyeongsan, Gyeongsangbuk-do, South Korea. Forty pregnant sows at 85 days of gestation were carefully selected. The sows, belonging to a three-way crossbreed of Landrace, Yorkshire, and Duroc breeds, were utilized for the experiment. The primiparous sows had an average weight of 160 ± 10 kg at 1 year old, while the multiparous sows had an average weight of 210 ± 10 kg at 3 years old. The sample population consisted of 20 primiparous sows and 20 multiparous sows, with the latter belonging to the 3rd parity range. Before administering the experimental treatment, an equal number of primiparous and multiparous sows among the pregnant population were randomly assigned to the control and treatment groups (Figure 1).

The animals were housed in sow pens equipped with proper ventilation and temperature control systems. The temperature was maintained between 18 °C and 22 °C (64 °F and 72 °F), with humidity levels kept between 40% and 70%. Each pen provided sufficient space for the sows to move freely and access feed and water easily. The flooring, made of concrete, featured proper drainage to uphold hygiene standards. This study was approved by the Institutional Animal Care and Use Committee (IACUC: GNU-170227-P0005) of Gyeongsang National University, Republic of Korea.

### 2.2. Experimental Treatment

For the experimental treatment, the sows in the treatment group received an intramuscular injection to induce short-term anesthesia of ketamine hydrochloride (Yuhan Co., Ltd., Seoul, Republic of Korea) at a dosage of 1.1 mg/kg body weight, along with telazol (Zoetis Korea, Seoul, Republic of Korea) at a dosage of 1.85 mg/kg. Subsequently, the test substance containing GHRH plasmid at a concentration of 2.5 g/mL (LifeTide SW5, VGX Animal Health, Woodlands, TX, USA) was injected into the semimembranous leg muscle of the hind limb using a 21-gauge hypodermic needle. The injection site was subjected to electrical stimulation using a CELLECTRA electroporation device (VGX Pharmaceuticals, Blue Bell, PA, USA) to facilitate uptake of the drug into cells. The electrical stimulation parameters for the electroporation device were 0.5 A, five pulses, with a duration of 52 ms per pulse, and a 1 s interval between pulses. The control group underwent the same procedure as the treatment group. However, instead of the 5 mg dose of GHRH plasmid, a 2 mL phosphate buffer solution (PBS) was injected into the semimembranosus leg muscle of the hind limb.

### 2.3. Concentration of IGF-1 in the Serum

Following weaning, blood samples were meticulously obtained from all sows through the jugular vein. To ensure optimal conditions for coagulation, the blood was allowed to clot naturally at room temperature for approximately 2 h. After clotting, the samples were centrifuged at 3000 rpm for 15 min, and the supernatant was collected for assaying. The serum collection was performed immediately and it was stored at −70 degrees until reanalysis. The isolated and purified serum was used for the measurement of IGF-1 concentration using a pig IGF-1 ELISA kit (LSBio in Shirley, MA, USA).

### 2.4. Sow Reproductive Indicators of Total Litter Size, Surviving Litter Size, Stillborn, and Birthweight

Sow reproductive indicators (total number of born piglets, number of live-born piglets, number of stillborn piglets, and birth weight of surviving piglets) were examined at farrowing for all sows. In addition, the birth weight of the surviving piglets was measured.

### 2.5. Number of Weaned Piglets and Weaned Piglet Weight

At the age of 4 weeks, weaning was initiated for all of the suckling piglets. During the weaning period, detailed records were maintained, which included the count and weight of piglets transitioning from maternal care, the number of weaned piglets, weaning rate, and weaning weight in the control and treatment groups injected with GHRH plasmid.

### 2.6. Statistical Analysis

The primiparous sow groups were compared with the multiparous sows. In addition, the GHRH-T groups were compared with the controls. Data were analyzed using GraphPad Prism software (version 8.0.1; Boston, MA, USA). Significant differences between groups were calculated by one-way factorial analysis of variance (ANOVA), followed by a Dunnett’s multiple comparisons test, and *p* < 0.05 was considered statistically significant.

## 3. Results

### 3.1. IGF-1 Concentration in Sows

**Figure 2:**
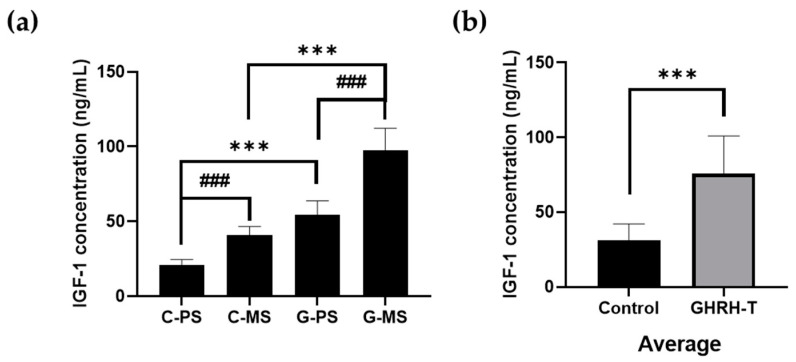
Effects of GHRH plasmid treatment on serum IGF-1 concentration in sows. (**a**) IGF-1 concentration of sow group (**b**) Average of IGF-1 concentration average. C-PS: Control primiparous sows, C-MS: Control multiparous sows, G-PS: GHRH-administered primiparous sows, G-MS: GHRH-administered multiparous sows. *** *p* < 0.001, compared with the control group. ^###^ *p* < 0.001, compared with each group.

In the control group, primiparous sows (C-PS) and multiparous sows (C-MS) exhibited a significant increase in serum IGF-1 concentration (*p* < 0.001). Furthermore, within the treatment group, primiparous sows (G-PS) and multiparous sows (G-MS) showed a significant increase in serum IGF-1 concentration compared to the GHRH-T group (*p* < 0.001). In the GHRH-T group, the serum IGF-1 concentration was significantly increased compared with the control group (*p* < 0.001). In addition, both primiparous sows (G-PS) and multiparous sows (G-MS) in the treatment group exhibited a significant increase in serum IGF-1 concentration compared with their respective control groups (*p* < 0.001). Following GHRH-T therapy, the significance of the increase in IGF-1 concentration across different sow parity statuses was determined (Figure 2 and Table 1).

### 3.2. The Number of Total Born Offspring, the Number Live-Born Offspring, the Number of Stillborn Offspring, and the Weight of the Sows

**Figure 3:**
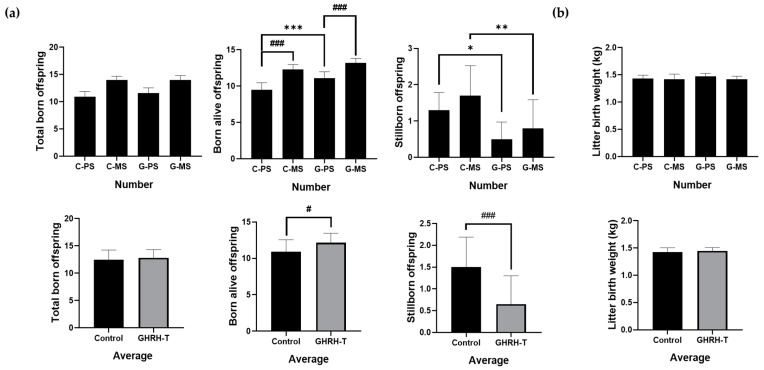
Effects of GHRH-T treatment on reproductive outcomes in primiparous and multiparous sows. (**a**) The reproductive outcomes were assessed by evaluating the total number of offspring produced, the number of offspring born alive, and the number of offspring born dead. Additionally, the sums and averages of these figures were calculated to provide a comprehensive overview of the reproductive results. (**b**) The evaluation also encompassed litter birth weight (kg) and the average total birth weight. C-S: Control primiparous sows, C-MS: Control multiparous sows, G-PS: GHRH-administered primiparous sows, G-MS: GHRH-administered multiparous sows. * *p* < 0.05, ** *p* < 0.01, *** *p* < 0.001, compared with the control group. ^#^ *p* < 0.05, ^###^ *p* < 0.001, compared with each group.

Following GHRH-T, significant improvements were observed in various reproductive outcomes compared to the control group. Although the total number of births increased, no significant difference was noted. However, the number of surviving piglets significantly increased (*p* < 0.001). Additionally, in the control group, both C-PS and C-MS showed a significant increase in the number of offspring born alive (*p* < 0.001). Moreover, within the treatment group, G-PS and G-MS demonstrated a significant increase in the number of offspring born alive compared to the GHRH-T (*p* < 0.001). Furthermore, a decrease in the number of stillborn piglets was observed, which was also statistically significant (*p* < 0.05, *p* < 0.01) (Figure 3a). However, no significant difference was found in birth weight between the control and treatment groups (Figure 3b).

Upon further examination of the primiparous sows in the treatment group (G-PS), marked enhancements in reproductive performance were evident. The number of surviving piglets (*p* < 0.001) was significantly increased compared with their counterparts in the control group (C-PS). Furthermore, a significant decrease in the number of stillborn piglets was observed in the treatment group (*p* < 0.05).

For multiparous sows in the treatment group (G-MS), an increase in the number of surviving piglets was observed, which was compared with the control group (C-MS). Moreover, a significant reduction in the number of stillborn piglets was observed (*p* < 0.05). In the treatment group, the birth weights of the piglets from primiparous and multiparous sows were not significantly different compared with the control group (Table 2).

### 3.3. Weaned Piglets and Weaned Piglet Weight Measurements

In the control group, primiparous sows (C-PS) and multiparous sows (C-MS) exhibited significant increases in the number of live-born offspring and the number of weaned piglets (*p* < 0.001). Additionally, within the treatment group, primiparous sows (G-PS) and multiparous sows (G-MS) showed significant increases in the number of live-born offspring and the number of weaned piglets when compared to the GHRH-T group (*p* < 0.001).

**Figure 4:**
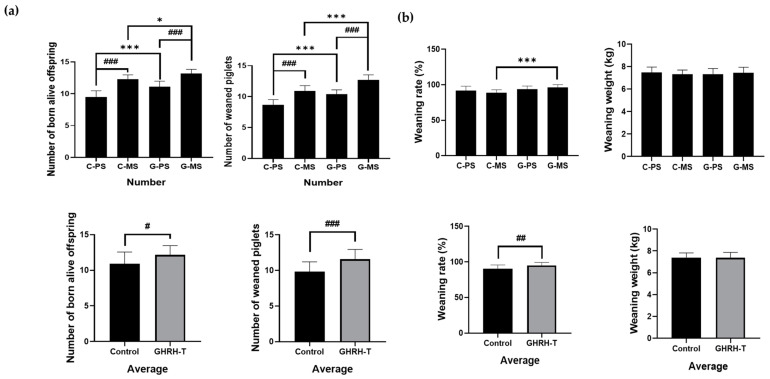
Effects of weaning piglet in primiparous and multiparous sows treated with GHRH-T. (**a**) Impact and average of GHRH-T on live births and weaned piglets in sows and (**b**) GHRH treatment on analyzing rates and weights in the litter. C-PS: Control primiparous sows, C-MS: Control multiparous sows, G-PS: GHRH-administered primiparous sows, G-MS: GHRH-administered multiparous sows. * *p* < 0.05, *** *p* < 0.001, compared with the control group. ^#^ *p* < 0.05, ^##^ *p* < 0.01 ^###^ *p* < 0.001 compared with each group.

In the GHRH-T group, significant improvements were observed in key indicators of piglet weaning outcomes compared with the control group. The total number of weaned piglets was significantly increased (*p* < 0.001), accompanied by a notable improvement in the weaning rate (*p* < 0.01). Although the weaned piglet weight in the treatment group exhibited a slight increase compared with that in the control group, this difference was statistically significant. An analysis of the outcomes specific to primiparous sows in the treatment group (G-PS) revealed a considerable enhancement in the total number of weaned piglets compared with their counterparts in the control group (C-PS) (*p* < 0.001); however, the weaning rate and weaned piglet weight were not significantly different compared with the control group (Figure 4a).

For multiparous sows in the treatment group (G-MS), significant improvements were observed in both the total number of weaned piglets and the weaning rate compared with the control group (C-MS) (*p* < 0.001). Similar to the findings in primiparous sows, the weaned piglet weight did not show a statistically significant difference compared with the control group (Figure 4b and Table 3). The significant increase in the total number of weaned piglets and weaning rate, particularly among primiparous and multiparous sows, suggests the potential benefits of GHRH-T in enhancing overall reproductive success and piglet production.

## 4. Discussion

GHRH is a peptide hormone primarily synthesized in the hypothalamus and released from the anterior pituitary gland [13]. It plays an important role in regulating the synthesis and secretion of GH from the pituitary gland. GH, in turn, exerts its effects on various tissues and organs primarily by stimulating the production of IGF-1. IGF-1 is predominantly produced in the liver and is an important mediator of the growth-promoting actions of GH [14]. It influences cellular growth, proliferation, differentiation, and metabolic processes in diverse tissues throughout the body. The relationship between GHRH and IGF-1 is integral to the regulation of growth [7].

This sequence represents an important axis involved in growth regulation, in which GH acts as a stimulator to produce IGF-1 to mediate many of the growth-promoting effects attributed to GH. IGF-1, which is primarily secreted by the liver in response to GH, serves as the main peripheral mediator of GH action [15,16]. This means that IGF-1 plays a crucial role in transmitting the biological signals initiated by GH to various target tissues and organs. In the present study, the administration of GHRH stimulated growth hormone secretion compared with the untreated control group. Moreover, it was observed that in sows with a history of parturition treated with GHRH-T, the level of IGF-1 increased by 56.33 ng/mL, and in non-parous sows, the level of IGF-1 increased by 33.27 ng/mL (Figure 2 and Table 1). These results suggest that GHRH effectively stimulates growth hormone secretion, which consequently affects the increase in IGF-1 levels observed in sows that have undergone parturition. GHRH is involved in an important regulatory system that influences growth, development, and the physiological processes associated with pregnancy and lactation. [17,18]. The benefits of increased maternal GHRH production on offspring survival and growth are believed to occur through various pathways.

Table 2 shows that the group administered with GHRH exhibited an increase in the total number of offspring among nulliparous sows, whereas no change was observed in multiparous sows. In addition, within the GHRH group, the survival rate of offspring increased uniformly, whereas the rate of stillbirths decreased. The effects of heightened maternal GHRH production on offspring survival and growth are likely attributed to a combination of various factors [19]. The administration of GHRH suggested potential implications for improving the uterine environment [20] and affecting the growth and development of conceptuses in sows.

The administration of the GHRH plasmid during pregnancy results in metabolic changes in the mother [21], which enhances nutrition consumption and breastfeeding, leading to more healthy offspring [6]. The results in Figure 4 and Table 3 reveal the notable impact of GHRH administration on the weaning phase in sows. In the GHRH-treated group, there was a significant increase in the total number of weaned piglets and the weaning rate compared with the control group, which indicated enhanced success in the weaning process. Notably, primiparous and multiparous sows in the treatment group exhibited heightened numbers of weaned piglets and improved weaning rates compared with their respective control groups. However, despite the increase in the total number of weaned piglets and weaning rates, weaned piglet weights between the treatment and control groups were similar. This suggests that while GHRH treatment positively influences the weaning success of piglets, it does not significantly impact the average weight of the weaned piglets. These outcomes suggest that the enhanced growth observed in the offspring of GHRH-treated sows before weaning is potentially linked to post-weaning effects on the pituitary component.

In the present study, we demonstrated that GHRH plasmid can directly affect the growing fetus [16,22]. GHRH plasmid may also act directly on the developing fetus, as we observed that GHRH protein can cross the placenta, impact the pituitary gland, increase somatotroph and lactotroph numbers, as previously reported, and increase the postnatal growth rate of the offspring. Overall, our results suggest that GHRH plasmid administration significantly influences serum IGF-1 levels and positively affects reproductive and postnatal outcomes in sows without significant effects on piglet weight at birth or weaning. These findings provide novel insight into swine management and breeding strategies within the agricultural sector.

## 5. Conclusions

The administration of the GHRH plasmid resulted in significant effects on various parameters. It increased serum IGF-1 levels in primiparous and multiparous sows, leading to improved birth results, such as increased total births, higher survival rates, and reduced stillbirths. Moreover, GHRH plasmid treatment positively influenced the weaning phase by increasing the total number of weaned piglets and the weaning rate. However, despite these effects, birth weights and weaned piglet weights were similar between the treatment and control groups. The GHRH plasmid is recognized for its pivotal physiological significance, and the outcomes of this study potentially have economic value to the pig farming sector.

## Figures and Tables

**Table 1:** Serum insulin-like growth factor-1 concentration in lactating sows immediately after weaning.

Group		IGF-1 Concentration (ng/mL)
Control	C-PS	20.91 ± 3.69
	C-MS	40.96 ± 5.63 ^###^
	Average	30.93 ± 11.28
GHRH-T	G-PS	54.18 ± 9.54 ***
	G-MS	97.29 ± 14.95 *** ^###^
	Average	75.74 ± 25.26 ***

Control was administered to the semimembranosus leg muscle with 2 mL of PBS using a 21-gauge hypodermic needle, followed by electroporation. GHRH-T was administered to the semimembranosus leg muscle with 5 mg of GHRH plasmid using a 21-gauge hypodermic needle, followed by electroporation. PS: primiparous sows, MS: multiparous sows. *** *p* < 0.001, compared with the control group. ^###^ *p* < 0.001, compared with each group.

**Table 2:** Total number of born, live-born, and stillborn offspring, as well as litter birth weights from control and treatment groups injected with GHRH-T.

Group		Number			Litter Birth Weight (kg)
		Total Born Offspring	Live-Born Offspring	Stillborn Offspring	
Control	C-PS	10.90 ± 0.99	9.50 ± 0.97	1.30 ± 0.48	1.43 ± 0.06
	C-MS	14.00 ± 0.67	12.30 ± 0.67 ^###^	1.70 ± 0.82	1.42 ± 0.10
	Average	12.45 ± 1.75	10.90 ± 1.65	1.50 ± 0.69	1.43 ± 0.08
GHRH-T	G-PS	11.60 ± 0.97	11.10 ± 0.88 ***	0.50 ± 0.53 *	1.48 ± 0.05
	G-MS	14.00 ± 0.82	13.20 ± 0.63 ^###^	0.80 ± 0.79 **	1.42 ± 0.05
	Average	12.80 ± 1.51	12.15 ± 1.31 *	0.65 ± 0.67 ***	1.45 ± 0.06

Control was administered to the semimembranosus leg muscle with 2 mL of PBS using a 21-gauge hypodermic needle, followed by electroporation. GHRH-T was administered to the semimembranosus leg muscle with 5 mg of GHRH plasmid using a 21-gauge hypodermic needle, followed by electroporation. PS: primiparous sows, MS: multiparous sows. * *p* < 0.05, ** *p* < 0.01, *** *p* < 0.001, compared with the control group. ^###^ *p* < 0.001, compared with each group.

**Table 3:** The number of weaning piglets, weaning rate, and weaning weight in the control and treatment groups injected with GHRH plasmid.

Group		Number of Live-Born Offspring	Number of Weaned Piglets	Weaning Rate (%)	Weaning Weight (kg)
Control	C-PS	9.50 ± 0.97	8.70 ± 0.82	91.82 ± 6.16	7.48 ± 0.48
	C-MS	12.30 ± 0.10 ^###^	10.90 ± 4.35 ^###^	88.57 ± 0.38	7.31 ± 0.38
	Average	10.09 ± 1.65	9.80 ± 1.40	90.20 ± 5.45	7.39 ± 0.43
GHRH-T	G-PS	11.10 ± 0.88 ***	10.40 ± 0.70 ***	93.85 ± 4.28	7.33 ± 0.51
	G-MS	13.20 ± 0.63 * ^###^	12.70 ± 0.82 *** ^###^	96.21 ± 4.00 ***	7.44 ± 0.50
	Average	12.15 ± 1.31 *	11.55 ± 1.39 ***	95.03 ± 4.21 **	7.38 ± 0.49

Control was administered to the semimembranosus leg muscle with 2 mL of PBS using a 21-gauge hypodermic needle, followed by electroporation. GHRH-T was administered to the semimembranosus leg muscle with 5 mg of GHRH plasmid using a 21-gauge hypodermic needle, followed by electroporation. PS: primiparous sows, MS: multiparous sows. * *p* < 0.05, ** *p* < 0.01, *** *p* < 0.001, compared with the control group. ^###^ *p* < 0.001, compared with each group.

## Data Availability

The data presented in this study are available upon request from the corresponding author.
